# MetaBIDx: a new computational approach to bacteria identification in microbiomes

**DOI:** 10.20517/mrr.2024.01

**Published:** 2024-04-01

**Authors:** Diem-Trang Pham, Vinhthuy Phan

**Affiliations:** Department of Computer Science, University of Memphis, Memphis, TN 38152, USA.

**Keywords:** Bacteria identification, metagenomics, species identification, bloom filter, clustering

## Abstract

**Objectives:** This study introduces MetaBIDx, a computational method designed to enhance species prediction in metagenomic environments. The method addresses the challenge of accurate species identification in complex microbiomes, which is due to the large number of generated reads and the ever-expanding number of bacterial genomes. Bacterial identification is essential for disease diagnosis and tracing outbreaks associated with microbial infections.

**Methods:** MetaBIDx utilizes a modified Bloom filter for efficient indexing of reference genomes and incorporates a novel strategy for reducing false positives by clustering species based on their genomic coverages by identified reads. The approach was evaluated and compared with several well-established tools across various datasets. Precision, recall, and F1-score were used to quantify the accuracy of species prediction.

**Results:** MetaBIDx demonstrated superior performance compared to other tools, especially in terms of precision and F1-score. The application of clustering based on approximate coverages significantly improved precision in species identification, effectively minimizing false positives. We further demonstrated that other methods can also benefit from our approach to removing false positives by clustering species based on approximate coverages.

**Conclusion:** With a novel approach to reducing false positives and the effective use of a modified Bloom filter to index species, MetaBIDx represents an advancement in metagenomic analysis. The findings suggest that the proposed approach could also benefit other metagenomic tools, indicating its potential for broader application in the field. The study lays the groundwork for future improvements in computational efficiency and the expansion of microbial databases.

## INTRODUCTION

Advances in next-generation sequencing technologies have reduced both cost and sequencing errors, enabling large-scale analyses of metagenomic data^[1]^ to help understand the microbial composition of environments like the human gut. This understanding can provide insights into various disorders and diseases^[2,3]^ including diabetes^[4,5]^, depression^[6,7]^, rheumatoid arthritis^[8]^, and gout^[9]^. Dysbiosis, or microbial imbalance, is not only linked to gastrointestinal disorders^[10]^ but can also affect the respiratory system^[3]^.

Key processes for analyzing microbial communities include read classification, profiling, and species identification. Read classification uses computational algorithms and a reference database to assign metagenomic reads to specific groups or organisms. Profiling assesses the relative abundance of different organisms in a sample, providing crucial environmental insights^[11-13]^. Species identification, particularly important in clinical metagenomics, determines the organisms present in a sample and is essential for diagnosing infections caused by specific pathogens. Despite their importance, these processes can be challenging due to the vast amount of information they require.

Various techniques exist for metagenomic analysis, including read alignment to reference genomes^[14]^, using taxonomically informative gene marker analysis^[15]^, clustering metagenomic sequence^[16-18]^, assembling sequence^[19,20]^, using unique characteristics of the 16S rRNA genes^[16,21]^, and using *k*-mers^[22-25]^. Alignment-based approaches are accurate but time-consuming, while *k*-mer-based approaches achieve a better balance between performance and runtime^[26]^. CLARK^[24]^, a read classifier, assigns reads to targets with the most distinguishing *k*-mers, and stands out for its efficiency and speed in read classification, making it suitable for extensive datasets. DUDes^[14]^, a taxonomic profiler, identifies candidate organisms by comparing read mapping strength in each node of the taxonomic tree iteratively, and demonstrates effectiveness in single and multiple organism detection, excelling in scenarios with unevenly represented references. Kraken^[25]^ creates a database with *k*-mers and corresponding common ancestors, then utilizes exact-match database queries of *k*-mers for rapid processing. Kraken achieved high precision and sensitivity at the genus level while also standing out for its accuracy and speed. MetaCache^[23]^, a *k*-mer based read classifier, uses a technique known as minhashing and context-aware *k*-mer matches, significantly reducing memory requirements while maintaining high sensitivity and precision. GSM^[22]^, another profiler, builds an index using genomic markers and computes abundances with linear equations, also showing its high accuracy in comparison to other tools. Several approaches utilize a specialized data structure for information retrieval known as a Bloom filter. FACS^[27]^ uses a Bloom filter to classify DNA sequences. MetaProFi^[28]^ uses a Bloom filter to build indexes of amino acid sequences to provide a memory-efficient and storage-efficient solution for protein sequence comparison. Bloom filters have recently been used to index large collections of short-read sequencing data. BIGSI^[29]^ and COBS^[30]^ use multiple Bloom filters to index *k*-mers in a way that attempts to limit cache misses during query. Kmtricks^[31]^ also used Bloom filters to index terabase-sized collections of sequencing data.

Most methods rely on read classification for species identification^[24,25]^. This process typically involves mapping sequenced reads against a reference database or index, and assigning reads to species based on how they are matched to the database or index. This approach, however, faces challenges caused by sequencing errors, mutations, horizontal gene transfer, or strain-level variation, which impact species identification. For instance, horizontal gene transfer can result in shared genetic segments across distinct species, complicating the attribution of reads to specific genomes. To mitigate this challenge, alternative strategies like metagenomic assembly can be employed^[19,20]^. This approach involves assembling reads into longer contiguous sequences, providing more contextual information than individual reads and aiding in more accurate species identification. This approach, however, has its own challenges. First, they are computationally demanding, requiring significant processing power and memory, especially for complex or large metagenomic datasets. Further, their success is heavily dependent on the quality and length of reads. Short or poor-quality reads may lead to fragmented assemblies, reducing the ability to reconstruct complete genomes. Lastly, assembling genomes of low-abundance species might create an unreliable assembly, leading to an underrepresentation of less abundant members of the microbial community.

Many existing approaches for species detection in metagenomics rely on the outcomes of read classification, which, while common, may not optimize species identification accuracy. This paper posits that prioritizing species identification directly enhances accuracy by providing a more precise representation of the microbiome community. We introduce a novel species identification method for microbiomes that utilizes distinctive genomic signatures and a modified Bloom filter for indexing the genomes within a microbiome. To reduce false positives and enhance identification accuracy, we integrate a clustering approach, an unsupervised machine learning technique. This method groups species with similar genomic coverages, facilitating the identification of species with low coverages that might otherwise be mistaken as artifacts due to inaccurate read detection. Our results show that this method outperforms existing techniques in terms of accuracy and successfully identifies a pathogen in an actual metagenomic dataset.

## METHODS

MetaBIDx, our proposed method, consists of two stages:

1. Index phase. This phase involves collecting reference genomes for a target microbiome and constructing an index from these reference genomes. The reference genomes represent the universe of species that may exist in a specific microbiome. The index comprises signatures of all *k*-mers (short sequences of length *k*) from potentially hundreds to thousands of reference genomes.

2. Prediction phase. Here, metagenomic DNA sequences, consisting of short reads from a metagenomic sample, are matched against the index, built in the first phase, to ascertain their probable species origins. This sample of reads comes from a specific environment or host containing species that is a subset of the universe of species, whose genomes are collected in the first phase. Subsequently, species of identified reads are clustered based on their approximate coverages, aiming to filter out false positive species predictions, leaving only the species present in the metagenomic environment.

### Phase 1: Building the index of a microbiome

MetaBIDx employs a modified Bloom filter^[32]^ as its indexing mechanism. A Bloom filter is a space-efficient probabilistic data structure used for efficient membership queries. Although a Bloom filter may mistakenly identify items not stored in it (false positive mistakes), it can accurately recall stored items.

Index construction for a microbiome relies on the reference genomes of species present. Without prior knowledge of the microbiome’s composition, one can use comprehensive bacterial and viral genomes from public databases for index creation. However, if specific species within the microbiome are of interest or there is partial knowledge about its composition, the index can be built using only those species’ reference genomes.

In constructing the index **F** for referenced bacterial genomes, each genome’s *k*-mers are processed. This includes both the main and reverse complement strands of a genome. A set of n randomly generated hash functions maps each *k*-mer to n entries in the index. These entries can hold three types of values: 0 for an empty entry, -1 for a “dirty” entry, and a positive genome id. An entry in **F** has value -1 if two *k*-mers from different genomes hash to it, indicating the *k*-mer is not unique. If a *k*-mer from one genome hashes to an entry already holding a different genome’s id, that *k*-mer is not unique, and the entry is marked as “dirty”. Otherwise, the entry is updated with the genome id of the currently processed genome if it is empty or already contains the same genome id.

This approach allows **F** to function similarly to a Bloom filter, aiding in the detection of genomes present in a metagenomic sample. The algorithm for building this index processes each genome sequentially, updating the index entries accordingly.

### Phase 2: Determining species in a microbiome

The process consists of two steps. First, the index **F** is used to assign reads in the sample to the species stored in the index. Second, these reads are further clustered into groups of similar coverages to determine which species are present in the sample.

#### Step 1: Querying reads

To determine which species a read belongs to, hash values of all *k*-mers within the read are checked against the index **F**. For a read that is part of genome g, if it contains a *k*-mer with a unique hash value in **F**, it is correctly identified as belonging to genome g. A read not belonging to genome g might be mistakenly identified as such if it contains a *k*-mer with a hash value matching one from genome g. This could be due to sequencing errors or genetic variants.

The strategy for querying each *k-*mer of a read is as follows. First, gather a set of values stored in **F**. If they consist of an identical positive value, we predict this value to be the id of the genome containing the *k*-mer. Otherwise, the *k*-mer is discarded.

If over 50% of non-discarded *k*-mers in a read have the same value, we predict this value to be the id of the genome that contains the read. Otherwise, the read is discarded. We adhere to the standard majority rule, setting the threshold at 50% as a default. Users, however, can adjust this threshold to desired stringency levels. After processing all reads in the sample, only those with predictively identified species are retained. The output includes these reads and their corresponding genome ids. This strategy helps to ensure accuracy in species identification despite potential errors or variants in the sequencing data.

#### Step 2: Identification of species based on approximate genomic coverages

Our method for predicting bacterial presence in a microbiome innovatively employs clustering based on the “approximate” coverages of bacterial genomes. Central to this approach is the identification of reads containing unique genetic signatures indicative of specific bacterial species. In this context, a bacterium’s presence is inferred from nontrivial genomic coverage, while an absence is suggested by minimal coverage, primarily due to false positives. The underlying assumption of our approach is that with modern sequencing technologies, there will be fewer sequencing errors, yielding fewer false positives. Consequently, falsely predicted species caused by falsely predicted reads should have significantly low coverages.

In this step, species with similar “approximate” coverages are placed in the same clusters. “Approximate” coverages are calculated based on the number of identified reads, which contain unique *k*-mers of species. A crucial step involves identifying and examining the cluster with the lowest mean coverage. This cluster comprises species with exceptionally low coverages, attributable to misidentified unique signatures. By excluding these species from our predictions, we significantly reduce the incidence of false positives. We employed a popular clustering method, K-means, to cluster species with similar coverages. The implementation of K-means was provided by scikit-learn^[33,34]^.

### Data collection and preparation

To evaluate the proposed method, we employed metagenomic shotgun sequencing data without imposing data quality constraints or specific requirements. Three widely recognized datasets were utilized for this assessment, comprising two mock community datasets and one derived from a human sample.

Mende Dataset: this dataset (available at https://swifter.embl.de/~mende/simulated_data), comprises three metagenomic samples. These samples are distinguished by their species complexity, featuring 10, 100, and 400 species, respectively. Each sample contains 75 bp long reads. The number of reads varies from 26,665,674 to 26,667,004 pairs. This dataset, originally used in a study on metagenomic assembly^[35]^, was constructed using simulated Illumina sequencing errors and quality values, reflecting the characteristics of actual metagenomic data.

CAMI Challenge Dataset: this dataset was obtained from the CAMI challenge^[36]^ (accessible at https://data.cami-challenge.org). It was also used in another benchmark^[37]^. It includes eight metagenomic samples representing a gradient of complexity: low (RL_S001), medium (RM_S001, RM_S002), and high (RH_S001, RH_S002, RH_S003, RH_S004, RH_S005). These samples are characterized by experimental conditions and features akin to real datasets, such as the inclusion of multiple, closely related strains, the presence of plasmid and viral sequences, and realistic abundance distributions. The reads in this dataset are 150 bp in length. The number of reads varies from 49,898,179 to 49,905,935 pairs, from low complexity to high complexity.

PT-8 (S2): this sample was used in a study^[38]^. It was derived from brain tissue biopsies of a 67-year-old patient with osteomyelitis, lung disease, and multifocal brain and spinal lesions, and was diagnosed with *Mycobacterium tuberculosis*. The PT-8 (S2) sample, with human reads excluded, can be accessed at the NCBI SRA repository (https://www.ncbi.nlm.nih.gov/sra/SRX1621515). This sample consists of bacterial, viral, and fungal species.

Reference Genomes: methods that use reference genomes to build indices for species identification. For the Mende dataset, we collected reference genomes that contain reads from all four metagenomic samples. This resulted in a set of 457 bacterial genomes, averaging approximately 3.5 Mbp each, with a cumulative size of around 1.6 GB. In contrast, the reference genomes for the CAMI dataset were more extensive, encompassing 2,850 bacterial genomes. This collection included all bacterial genomes present in the reads from the eight samples, along with genomes of closely related strains within the same species or subspecies. The average genome size in this collection was about 5.7 Mbp, culminating in a total size of approximately 16 GB. The genome collection for the CAMI dataset was utilized in the experiment with real dataset as it also contains *Mycobacterium tuberculosis*. All reference genomes were downloaded from NCBI.

### Comparative analysis of different methods for species prediction

We conducted a comprehensive comparative analysis of our tool, MetaBIDx, and various metagenomic tools, which can be used for species detection, including CLARK^[24]^, Kraken2^[39]^, KrakenUniq^[40]^, Centrifuge^[41]^, and Sourmash^[42]^. These tools were selected based on their robustness, documented accuracy, processing speed, and the ability to create custom indexes, a vital feature for our analysis. Tools that lacked comprehensive documentation or presented installation and experimental challenges were not considered for this study.

After experimenting with several *k*-mer lengths, MetaBIDx was built with *k*-mers of length 31. A previous study^[43]^ experimented with different *k*-mer sizes and observed that *k*-mer similarity between genomes approximated various degrees of taxonomic similarity, and that a *k*-mer length of 31 appeared to correspond to species-level similarity. Most tools used in the evaluation also have the default *k*-mer size of 31. An index with a size of 8 GB, utilizing 3 hash functions, was created for the Mende dataset, which included 457 reference genomes. For the CAMI dataset, a more extensive index of 16 GB was built using 2 hash functions, accommodating 2,850 reference genomes. The other tools in the study also employed *k*-mers of length 31 and were run with their default settings for a fair comparison. To ensure consistency across all methods, the same collections of reference genomes were used to construct the genome libraries or indexes. The experiments were conducted on a standardized computational setup, using a machine with 32 cores and 330 GB of RAM, and all tools were run in multi-threaded mode to utilize the full computational capacity. The script for building index for all tools is shared in the Supplementary Materials.

The evaluation of prediction performance utilized three widely recognized metrics: precision, recall, and F1-score. Precision quantifies the proportion of true positive predictions out of all positive predictions made (sum of true positives and false positives), essentially reflecting the accuracy in predicting species as a fraction of all species predictions. Recall measures the proportion of true positive predictions relative to the total actual species present in the sample, indicating the method’s ability to identify all relevant species. The F1-score, derived as the harmonic mean of precision and recall, offers a composite metric that equally weights precision and recall, providing a single measure to assess the balance between them.

The comparative assessment of our method, MetaBIDx, alongside other tools, was structured into two key experiments. The first experiment focused on evaluating the ability of MetaBIDx and other tools to predict bacterial species based on identified reads alone, which is the standard approach adopted by these tools for species prediction in metagenomic samples. This experiment’s main objective was to show that the default behavior of these tools could be enhanced for more accurate species identification.

In the second experiment, we aimed to facilitate a more equitable comparison by augmenting the other methods with our strategy for reducing false positives. This was achieved by applying our technique of clustering “approximate” coverages to each tool. The inclusion of this approach in the assessment was intended to improve the precision of the other tools, thereby enabling a fairer and more balanced comparison with MetaBIDx. Through this two-pronged experimental design, we sought to comprehensively evaluate and demonstrate the effectiveness of our method in the context of metagenomic species prediction.

Additionally, we also explored the effect of using high-quality *k*-mers at different thresholds to enhance species prediction accuracy. This dual-phase approach was designed to provide a thorough understanding of the capabilities and limitations of each tool in metagenomic species identification.

## RESULTS

### Comparative analysis of species identification

First, we compared MetaBIDx against other methods that predict species solely based on read classification. This approach predicates species prediction on the detection of reads originating from the species in question. If a read from a particular species is detected in a sample, that species is predicted to be present. Such methods rely on the accuracy of reads identification to determine the presence or absence of species in a sample.

Mende Dataset. Table 1 reports the performance of our method versus the others on the Mende dataset. The comparison clearly demonstrates that MetaBIDx significantly outperforms other tools in predicting species across different sample complexities.

**Table 1:** Comparison of species identification on Mende and CAMI datasets

**Dataset**	**Sample**	**Method**	**Precision**	**Recall**	**F1-score**
Mende	10 species	MetaBIDx	**1.000**	0.800	0.889
		CLARK	0.027	**1.000**	0.053
		KrakenUniq	0.040	0.800	0.075
		Kraken2	0.025	0.800	0.048
		Centrifuge	0.021	0.800	0.041
		Sourmash	0.900	0.900	**0.900**
	100 species	MetaBIDx	**1.000**	0.976	**0.988**
		CLARK	0.236	**1.000**	0.382
		KrakenUniq	0.234	0.713	0.352
		Kraken2	0.222	0.760	0.345
		Centrifuge	0.221	0.850	0.351
		Sourmash	0.852	0.750	0.800
	400 species	MetaBIDx	**1.000**	0.970	**0.985**
		CLARK	0.876	**1.000**	0.933
		KrakenUniq	0.952	0.651	0.773
		Kraken2	0.870	0.753	0.807
		Centrifuge	0.876	0.883	0.879
		Sourmash	0.837	0.849	0.843
CAMI	RH_S001	MetaBIDx	**0.862**	0.449	**0.591**
		CLARK	0.059	**1.000**	0.111
		KrakenUniq	0.054	0.498	0.097
		Kraken2	0.087	0.427	0.144
		Centrifuge	0.058	0.536	0.104
		Sourmash	0.286	0.539	0.374
	RH_S002	MetaBIDx	**0.843**	0.677	**0.751**
		CLARK	0.059	**1.000**	0.111
		KrakenUniq	0.054	0.498	0.097
		Kraken2	0.087	0.427	0.144
		Centrifuge	0.058	0.536	0.104
		Sourmash	0.287	0.550	0.377
	RH_S003	MetaBIDx	**0.885**	0.689	**0.774**
		CLARK	0.059	**1.000**	0.111
		KrakenUniq	0.054	0.498	0.097
		Kraken2	0.087	0.427	0.144
		Centrifuge	0.058	0.536	0.104
		Sourmash	0.274	0.550	0.366
	RH_S004	MetaBIDx	**0.839**	0.778	**0.807**
		CLARK	0.059	**1.000**	0.111
		KrakenUniq	0.054	0.498	0.097
		Kraken2	0.087	0.427	0.144
		Centrifuge	0.058	0.536	0.104
		Sourmash	0.296	0.550	0.385
	RH_S005	MetaBIDx	**0.854**	0.737	**0.791**
		CLARK	0.059	**1.000**	0.111
		KrakenUniq	0.054	0.498	0.097
		Kraken2	0.087	0.427	0.144
		Centrifuge	0.058	0.536	0.104
		Sourmash	0.273	0.550	0.365
	RM_S001	MetaBIDx	**0.821**	0.397	**0.535**
		CLARK	0.020	**1.000**	0.040
		KrakenUniq	0.019	0.573	0.036
		Kraken2	0.023	0.393	0.044
		Centrifuge	0.020	0.629	0.039
		Sourmash	0.400	0.607	0.482
	RM_S002	MetaBIDx	**0.941**	0.276	0.427
		CLARK	0.020	**1.000**	0.040
		KrakenUniq	0.019	0.573	0.036
		Kraken2	0.023	0.393	0.044
		Centrifuge	0.020	0.629	0.039
		Sourmash	0.519	0.629	**0.569**
	RL_S001	MetaBIDx	**1.000**	0.421	**0.593**
		CLARK	0.007	**1.000**	0.013
		KrakenUniq	0.006	0.654	0.013
		Kraken2	0.010	0.577	0.020
		Centrifuge	0.007	0.731	0.013
		Sourmash	0.388	0.731	0.569

In the 10-species sample, MetaBIDx achieved perfect precision (1.000) and a high recall (0.800), resulting in an F1-score of 0.889. Sourmash also achieved a competitive precision (0.900) and a high recall (0.900), as well as the highest F1-score of 0.900. This is markedly superior to the other classification tools, which, despite high recall values (0.800 for KrakenUniq, Kraken2, and Centrifuge, and 1.000 for CLARK), had very low precision, leading to considerably lower F1-scores (ranging from 0.041 to 0.075 for the two Kraken and Centrifuge tools, and 0.053 for CLARK).

In the 100-species sample, MetaBIDx again maintained perfect precision (1.000) and an increased recall (0.976), resulting in an F1-score of 0.988. Sourmash still had competitive precision (0.852), recall (0.750), and an F1-score of 0.800. The other tools showed improvement in precision compared to the 10-species sample but were still significantly lower than MetaBIDx. Their recall values ranged from 0.713 to 1.000, and F1-scores varied between 0.345 and 0.382, still considerably lower than MetaBIDx.

For the most complex sample with 400 species, MetaBIDx maintained its high performance with perfect precision (1.000) and a recall of 0.970, leading to an F1-score of 0.985. Other tools showed a notable improvement in this category, with CLARK reaching an F1-score of 0.933. However, MetaBIDx still outperformed them, as the F1-scores for KrakenUniq, Kraken2, Centrifuge, and Sourmash were 0.773, 0.807, 0.879, and 0.843, respectively.

Overall, MetaBIDx consistently exhibits superior performance in species prediction across various sample complexities, particularly in maintaining high precision without sacrificing recall, leading to significantly higher F1-scores compared to other tools.

CAMI dataset. Table 1 reports the performance of our method versus the others on the Mende dataset. The results highlight the comparative efficacy of these tools across a range of samples with varying complexities.

High complexity samples (RH_S001 to RH_S005): In these samples, MetaBIDx consistently demonstrated superior precision, ranging from 0.839 to 0.885, and recall values varied from 0.449 to 0.778. This resulted in F1-scores between 0.591 and 0.807, significantly higher than the other tools. In contrast, tools like CLARK, KrakenUniq, Kraken2, and Centrifuge exhibited much lower precision (consistently below 0.1) and F1-scores, despite having high recall values. This indicates that while these tools were able to identify a broad range of species (high recall), they also misidentified many species (low precision), reducing their overall accuracy. Sourmash’s F1-scores varied from 0.365 to 0.385, slightly higher than other tools.

Medium complexity samples (RM_S001 and RM_S002): MetaBIDx again outperformed the other tools with higher precision and F1-scores. Particularly in RM_S001, MetaBIDx achieved a high precision of 0.821, albeit with a lower recall of 0.397, resulting in an F1-score of 0.535. In RM_S002, Sourmash achieved the highest F1-score (0.569), followed by MetaBIDx (0.427). The other tools, while maintaining perfect or near-perfect recall, had very low precision and F1-scores, indicating a high rate of false positives in species identification.

Low complexity sample (RL_S001): MetaBIDx achieved perfect precision (1.000) and a recall of 0.421, leading to an F1-score of 0.593. This is substantially better than the other tools, which, despite having high recalls, had extremely low precision and F1-scores.

In summary, MetaBIDx consistently outperformed the other tools across the CAMI dataset, particularly in terms of precision and F1-score. This suggests that MetaBIDx is more effective in accurately identifying the species present in a sample, with fewer false positives compared to other methods. Its performance is particularly notable in complex samples, where accurate species identification is more challenging.

### Enhancing precision with clustering of “approximate” coverages

Here, we aim to present a more equitable comparison between our tool and the others. The inherent limitation of these read-classification methods lies in their tendency to generate a high number of false positives, leading to lower precision in species prediction, as observed in the previous section.

We applied the false-positive-reduction strategy to all methods. This strategy groups species with similar coverages into the same clusters. The rationale is that true positives (actual species present in the sample) will typically show higher coverage compared to false positives (species incorrectly identified due to random read matches). By filtering out species with low coverage, we aim to reduce the number of false positives, thereby increasing the precision of species identification across all methods.

In this experiment, we excluded Sourmash as this tool is not a read classification tool. The result is summarized in Table 2.

**Table 2:** Comparison of species identification based on *approximate* coverage on Mende and CAMI datasets

**Dataset**	**Sample**	**Method**	**Precision**	**Recall**	**F1-score**
Mende	10 species	MetaBIDx	**1.000**	**0.800**	**0.889**
		CLARK	**1.000**	**0.800**	**0.889**
		KrakenUniq	**1.000**	**0.800**	**0.889**
		Kraken2	**1.000**	0.700	0.824
		Centrifuge	0.471	**0.800**	0.593
	100 species	MetaBIDx	**1.000**	**0.976**	**0.988**
		CLARK	0.988	0.859	0.919
		KrakenUniq	0.982	0.644	0.778
		Kraken2	0.985	0.670	0.798
		Centrifuge	0.790	0.830	0.810
	400 species	MetaBIDx	**1.000**	**0.970**	**0.985**
		CLARK	0.990	0.965	0.977
		KrakenUniq	0.989	0.634	0.773
		Kraken2	0.987	0.733	0.841
		Centrifuge	0.949	0.882	0.915
CAMI	RH_S001	MetaBIDx	**0.862**	0.449	**0.591**
		CLARK	0.688	**0.515**	0.589
		KrakenUniq	0.673	0.258	0.373
		Kraken2	0.699	0.238	0.356
		Centrifuge	0.624	0.275	0.382
	RH_S002	MetaBIDx	**0.843**	0.677	**0.751**
		CLARK	0.650	**0.868**	0.744
		KrakenUniq	0.640	0.427	0.512
		Kraken2	0.630	0.384	0.477
		Centrifuge	0.520	0.480	0.499
	RH_S003	MetaBIDx	**0.885**	0.689	**0.774**
		CLARK	0.670	**0.814**	0.735
		KrakenUniq	0.674	0.393	0.497
		Kraken2	0.667	0.377	0.482
		Centrifuge	0.621	0.417	0.499
	RH_S004	MetaBIDx	**0.839**	0.778	**0.807**
		CLARK	0.661	**0.910**	0.766
		KrakenUniq	0.637	0.458	0.533
		Kraken2	0.610	0.394	0.479
		Centrifuge	0.580	0.467	0.517
	RH_S005	MetaBIDx	**0.854**	0.737	**0.791**
		CLARK	0.677	**0.814**	0.739
		KrakenUniq	0.676	0.403	0.505
		Kraken2	0.651	0.364	0.467
		Centrifuge	0.578	0.444	0.502
	RM_S001	MetaBIDx	**0.821**	0.397	**0.535**
		CLARK	0.686	**0.414**	0.516
		KrakenUniq	0.611	0.247	0.352
		Kraken2	0.577	0.169	0.261
		Centrifuge	0.558	0.270	0.364
	RM_S002	MetaBIDx	**0.941**	**0.276**	**0.427**
		CLARK	0.833	0.259	0.395
		KrakenUniq	0.800	0.135	0.231
		Kraken2	0.778	0.079	0.143
		Centrifuge	0.833	0.169	0.280
	RL_S001	MetaBIDx	**1.000**	**0.421**	**0.593**
		CLARK	**1.000**	**0.421**	**0.593**
		KrakenUniq	**1.000**	0.269	0.424
		Kraken2	0.889	0.308	0.457
		Centrifuge	**1.000**	0.308	0.471

Mende Dataset: Applying the clustering strategy significantly improved the precision of all methods, particularly in the 10-species sample, where MetaBIDx, CLARK, KrakenUniq, and Kraken2 all achieved perfect precision (1.000). MetaBIDx maintained its high performance across all samples, consistently showing high precision and recall, resulting in F1-scores ranging from 0.889 to 0.985. CLARK, KrakenUniq, and Kraken2 also showed notable improvements in precision and F1-scores compared to their performance without clustering, particularly in samples with 100 and 400 species.

CAMI Dataset: In the CAMI dataset, the application of clustering also enhanced precision for all methods. MetaBIDx consistently demonstrated high precision and recall, with F1-scores ranging from 0.535 to 0.807 across different samples. Other tools, including CLARK and KrakenUniq, exhibited considerable improvements in precision, leading to higher F1-scores compared to their initial performance without clustering. However, MetaBIDx maintained an edge in terms of overall accuracy.

In conclusion, by adopting the clustering of “approximate” coverage, all methods showed an increase in precision, thereby reducing false positives. This approach demonstrates that integrating coverage-based clustering can significantly enhance the accuracy of species prediction in metagenomic analysis. MetaBIDx, with its inherent design to utilize this technique, consistently outperformed or matched the performance of other tools under this enhanced comparison framework.

### Identification of pathogens in human samples

We evaluated the performance of all tools in identifying the pathogen in the human sample PT-8 (S2) dataset, at the species level using an index built from 2,850 reference genomes. This sample was diagnosed with a disease organism, which we assumed as the ground truth.

We found that MetaBIDx had the highest rate of identified reads at 83%, followed by Kraken2, KrakenUniq, and Centrifuge with similar rates. CLARK had the lowest rate of identified reads, only reaching 42%. All tools assigned approximately 70% of identified reads to *Mycobacterium tuberculosi*s and the remaining 30% to other species.

When clustering of species based on coverage derived from identified reads was used, all tools identified *Mycobacterium tuberculosis* as the predicted species. It is important to note that PT-8 (S2) was used in a prior study^[38]^ and was derived from brain tissue biopsies of a 67-year-old patient with osteomyelitis, lung disease, and multifocal brain and spinal lesions. The patient was diagnosed with *Mycobacterium tuberculosis* and responded promptly to anti-tuberculous treatment. This suggested that our approach to reducing false positives via clustering based on genome coverage was effective and could be clinically beneficial.

### The impact of using high-quality *k*-mers

Sequencing errors can lead to false positives, reducing the precision of species prediction. We evaluated the impact of *k*-mer quality on the accuracy of bacterial prediction using MetaBIDx. *K*-mer quality was determined by averaging the quality scores of its constituent bases. For the Mende dataset, thresholds of 33 (the lowest quality) and 49 (the median quality) were used; for the CAMI dataset, thresholds of 18 (the lowest quality) and 30 (the median quality) were used. MetaBIDx lets users adjust the *k-*mer quality parameter to a desirable level.

The findings, summarized in Table 3, indicate that higher quality thresholds for *k*-mers lead to improved precision in bacterial species identification. This improvement was particularly notable in the CAMI dataset, where a significant increase in precision was observed, although it was accompanied by a slight reduction in recall.

**Table 3:** Prediction performance with different *k*-mer quality thresholds

**Dataset**	**Sample**	**Quality**	**Precision**	**Recall**	**F1-score**
Mende	10 species	33	0.024	1.000	0.047
		49	0.024	1.000	0.047
	100 species	33	0.203	1.000	0.337
		49	0.203	1.000	0.337
	400 species	33	0.945	1.000	0.972
		49	0.945	1.000	0.972
CAMI	RH_S001	18	0.261	0.970	0.411
		30	0.532	0.850	0.654
	RH_S002	18	0.245	0.970	0.411
		30	0.549	0.880	0.676
	RH_S003	18	0.244	0.982	0.390
		30	0.527	0.868	0.656
	RH_S004	18	0.250	0.970	0.398
		30	0.535	0.868	0.662
	RH_S005	18	0.242	0.970	0.398
		30	0.500	0.886	0.639
	RM_S001	18	0.159	0.914	0.270
		30	0.402	0.707	0.513
	RM_S002	18	0.161	0.914	0.273
		30	0.441	0.845	0.580
	RL_S001	18	0.082	1.000	0.151
		30	0.367	0.947	0.529

Mende Dataset: For the 10 and 100 species samples, there was no change in precision and recall when the *k*-mer quality threshold was increased from 33 to 49. In the 400 species sample, both thresholds (33 and 49) resulted in high precision and recall, with an F1-score of 0.972.

CAMI Dataset: Across all samples, increasing the *k*-mer quality threshold from 18 to 30 led to a notable improvement in precision and F1-scores. The increase in *k*-mer quality, however, resulted in a decrease in recall, though the overall F1-score improvement suggests a favorable balance between precision and recall. The trade-off between precision and recall highlights the importance of selecting an optimal *k*-mer quality threshold that balances the need for accurate species identification and comprehensive species detection.

### Running time analysis

We reported the running time of MetaBIDx on building the Mende index and querying reads from 400 species sample using different numbers of CPU(s) in Supplementary Tables 1 and 2. The running time of building the Mende index decreases significantly from 165 to 65 min as the CPU increases from 1 to 32. Similarly, the running time of querying reads from 400 species sample also decreases significantly from 145 min with 1 CPU to 16 min with 32 CPUs. The results in Supplementary Tables 1 and 2 indicate the effectiveness of parallelization in reducing computational time.

The comparison of running times between our method, MetaBIDx, and other tools such as CLARK, KrakenUniq, Kraken2, and Centrifuge indicates that MetaBIDx generally has longer running times across different samples in both the Mende and CAMI datasets. For the Mende dataset, on average, for each sample, MetaBIDx took 16 min, CLARK took 6 min, Kraken took 2 min, KrakenUniq and Centrifuge took 7 min. For the CAMI dataset, the average running times are 71, 17, 17, 8, and 16 min for MetaBIDx, CLARK, KrakenUniq, Kraken2, and Centrifuge, respectively. Supplementary Table 3 reports detailed information on this comparison.

## DISCUSSION

The proposed method employs Bloom filters to store unique genomic signatures and facilitates species indexing. It incorporates a novel strategy for reducing false positives by clustering species based on their “approximate” coverages derived from identified reads. We found that the method surpassed several well-known metagenomic tools in precision, recall, and F1-score across various datasets, particularly in complex microbiomes where accurate species identification is vital.

The approach for reducing false positives based on clustering “approximate” genome coverages, notably enhances prediction precision of not only MetaBIDx but also other approaches based on read classification, representing an advancement in addressing the prevalent issue of false positives in metagenomic analysis.

The proposed approach has wide-ranging implications for diverse metagenomic applications, such as environmental monitoring, human microbiome research, and disease diagnostics. Its capability to accurately detect low-abundance species and differentiate closely related species is particularly valuable in these fields. The integration of MetaBIDx with other bioinformatics tools could lead to a more robust and comprehensive workflow in metagenomic analysis.

Future research and development will focus on enhancing computational efficiency, expanding the microbial database for broader coverage, and refining the algorithm for increased accuracy. The study acknowledges certain limitations, including the computational demands of MetaBIDx, especially with exceptionally large datasets. Future versions aim to address these challenges, enhancing scalability and efficiency for more complex microbiome datasets. A crucial factor for the current version’s slower performance compared to established methods is the implementation focus on correctness and bug reduction rather than optimization. Future iterations of MetaBIDx will prioritize code optimization to improve running times while maintaining high performance in species prediction.

Currently, MetaBIDx can identify and output unique *k*-mers for each reference genomes in the database. This functionality can be enhanced by enabling the tool to output unique regions. This upgrade will significantly improve the utility of our tool, particularly providing more precise genomic signatures for each genome, allowing for better discrimination between closely related organisms or strains.

While we currently focus on species-level prediction, there is potential for explorations at higher taxonomic levels, such as family, class, or genus. Although the current version does not exploit taxonomic tree structures, MetaBIDx can identify uncatalogued species not yet included in existing databases. This capability is particularly beneficial for studying unexplored microbial communities or those from understudied environments.

## DECLARATIONS

### Availability of data and materials

Code and sample data reported in this manuscript are publicly available on GitHub (https://github.com/pdtrang/MetaBIDx).

### Copyright

© The Author(s) 2024.
