# Supplementary material for: MetaBIDx: a new computational approach to bacteria identification in microbiomes
Source: Microbiome Res Rep. 2024 Apr 1;3(2):25. doi: 10.20517/mrr.2024.01 (PMC11149084; doi:10.20517/mrr.2024.01)
Supplement: Supplementary file 1 [file mrr-3-2-25-SupplementaryMaterials.pdf]

Pham *et al. Microbiome Res Rep* 2024;3:xx | http://dx.doi.org/10.20517/mrr.2024.01

Supplementary Materials

MetaBIDx: a new computational approach to bacteria identification in microbiomes

Diem-Trang Pham, Vinhthuy Phan

Department of Computer Science, University of Memphis, Memphis, TN 38152, USA.

Correspondence to: Dr. Vinhthuy Phan, Department of Computer Science, University of Memphis, Memphis, TN 38152, USA. E-mail: vphan@memphis.edu

ORCID: 0000-0001-6108-1228 (Vinhthuy Phan), 0000-0001-9521-4789 (Diem-Trang Pham)

Running Time Analysis

Supplementary Table 1 shows the running time in minutes of building MetaBIDx index for Mende dataset with different number of CPU(s)

Supplementary Table 1. Running time (minutes) of building MetaBIDx index for Mende dataset with different number of CPU(s)

Number of CPU(s)	Running time
1	164.25
4	100.19
8	90.38
16	81.18
32	64.19

Supplementary Table 2 shows the running time in minutes of querying reads on Mende 400 species sample with different number of CPU(s).

Supplementary Table 2. Running time (minutes) of read classification on Mende 400species sample with different number of CPU(s)

Number of CPU(s)	Running time
1	146.20
4	40.45
8	24.39
16	20.20
32	15.48

Supplementary Table 3 shows the running time in minutes of all the tools on each sample in Mende and CAMI dataset.

Supplementary Table 3. Running time (minutes) of read classification on Mende and CAMI dataset

Dataset	Sample	MetaBIDx	CLARK	KrakenUniq	Kraken2	Centrifuge
Mende	10species	15.52	4.35	5.14	2.54	6.36
	100species	15.33	5.38	5.45	2.11	6.13
	400species	15.48	5.34	7.21	1.57	6.23
CAMI	RH_S001	69.09	12.36	18.26	6.41	13.43
	RH_S002	69.28	12.38	18.55	6.49	19.45
	RH_S003	68.21	17.13	19.26	6.36	19.43
	RH_S004	70.17	17.16	19.29	6.38	19.45
	RH_S005	73.58	17.21	18.50	6.45	19.55
	RM_S001	68.44	17.41	13.55	6.44	9.38
	RM_S002	69.16	17.53	13.48	6.57	9.31
	RL_S001	68.59	17.32	10.58	7.22	9.32

Script for building index for all tools in the experiment

All tools were run with k-mer length of 31 and used all CPUs. Other parameters were in defaults. We followed the instructions of each tool to build the index.

MetaBIDx (https://github.com/pdtrang/MetaBIDx)

metabidx build –refseq references/Mende_genomes –k 31 –n 2 –save index/Mende_k31_2n.bin

CLARK (http://clark.cs.ucr.edu)

Pham *et al. Microbiome Res Rep* 2024;3:xx | http://dx.doi.org/10.20517/mrr.2024.01

CLARK loads and processes reference genomes during classification and doesn't have an extra step for index building.

Kraken2 (https://github.com/DerrickWood/kraken2)

We followed the steps to build custom database for Kraken2 from the manual (https://github.com/DerrickWood/kraken2/wiki/Manual#custom-databases)

for file in references/Mende_genomes/*.fa do
kraken2-build --add-to-library $file --db kraken2_customDB_Mende --kmer-len 31 --threads $(nproc) done

KrakenUniq (https://github.com/fbreitwieser/krakenuniq)

We followed the steps to build custom database for KrakenUniq from the manual (https://github.com/fbreitwieser/krakenuniq?tab=readme-ov-file#custom-databases-with-ncbi-taxonomy)

krakenuniq-build --db krakenuniq_customDB_Mende --kmer-len 31 --threads $(nproc) --taxids-for-genomes --taxids-for-sequences

Centrifuge (https://github.com/DaehwanKimLab/centrifuge)

centrifuge-build –conversion-table Mende_genomes.conv --taxonomy-tree Mende_genomes.tree --name-table Mende_genomes.name Mende_genomes.fa centrifuge_customDB_Mende_genomes

(conversion table, taxonomy tree, name table are followed the format described in http://www.ccb.jhu.edu/software/centrifuge/manual.shtml#custom-database)

Sourmash (https://github.com/sourmash-bio/sourmash)

sourmash sketch dna -p k=31,scaled=1000,noabund references/Mende_genomes/*.fa -o sourmash_Mende_genomes.sig
